# The reproductive advantages of a long life: longevity and senescence in wild female African elephants

**DOI:** 10.1007/s00265-015-2051-5

**Published:** 2016-01-19

**Authors:** Phyllis C. Lee, Victoria Fishlock, C. Elizabeth Webber, Cynthia J. Moss

**Affiliations:** Amboseli Trust for Elephants, P.O. Box 15135, Langata, 00509 Nairobi Kenya; Behaviour and Evolution Research Group, Psychology, School of Natural Sciences, University of Stirling, Stirling, FK9 4LA UK

**Keywords:** Age-specific reproductive rates, Care allocation, Mortality filters, Post-reproductive survival, Grandmothering

## Abstract

Long-lived species such as elephants, whales and primates exhibit extended post-fertile survival compared to species with shorter lifespans but data on age-related fecundity and survival are limited to few species or populations. We assess relationships between longevity, reproductive onset, reproductive rate and age for 834 longitudinally monitored wild female African elephants in Amboseli, Kenya. The mean known age at first reproduction was 13.8 years; only 5 % commenced reproduction by 10 years. Early reproducers (<12.5 years) had higher age-specific fertility rates than did females who commenced reproduction late (15+ years) with no differences in survival between these groups. Age-specific reproductive rates of females dying before 40 years were reduced by comparison to same-aged survivors, illustrating a mortality filter and reproductive advantages of a long life. Overall, 95 % of fertility was completed before 50, and 95 % of mortality experienced by age 65, with a mean life expectancy of 41 years for females who survived to the minimum age at first birth (9 years). Elephant females have a relatively long period (c. 16 years) of viability after 95 % completed fertility, although reproduction does not entirely cease until they are over 65. We found no evidence of increased investment among females aged over 40 in terms of delay to next birth or calf mortality. The presence of a mother reproducing simultaneously with her daughter was associated with higher rates of daughter reproduction suggesting advantages from maternal (and grandmaternal) co-residence during reproduction.

## Significance

Elephants exhibit extended post-fertile survival, although how fecundity changes over the 60+ years of the lifespan is known for only a few populations. We present data for 834 wild female African elephants which have been monitored for over 40 years. We test whether reproductive onset affects reproductive output and longevity, and assess whether older females (aged >40 years) follow strategies for producing and rearing calves that are specific to later life. We explore the effects of mothers’ later-life reproduction while living alongside daughters in a family. The patterns in this elephant population—a long life associated with greater success in reproduction and the importance of grandmothers to daughters—are discussed relative to other species that exhibit post-fertile survival. We evaluate potential selection pressures that may have resulted in long reproductive careers for female elephants.

## Introduction

Long-lived species tend to show trends in ageing and fertility that are distinct from those of rapidly reproducing, short-lived species (Reed et al. 2008; Hayward et al. 2014), but species at both ends of the life history continuum can exhibit prolonged post-reproductive lifespans with potential advantages for both the survivors and their offspring (Croft et al. 2015). The evolution of post-reproductive longevity thus remains a question of major theoretical interest. Recent analyses of age-specific rates of reproduction among wild primates (Alberts et al. 2013) found that the modal age of reproductive cessation coincided with modal ages of mortality and Asian elephants (*Elephas maximus*) exhibit a similar effect (Lahdenperä et al. 2014). By contrast and like humans, among killer whales (*Orcinus orca*) reproductive senescence occurs over 20 years before modal mortality (Foote 2008; Brent et al. 2015).

Elephants, humans and whales share a lifespan of 70–100+ years, making them rare among mammals. Maximum lifespan has been estimated at 74 years from tooth wear (Lee et al. 2012) in wild female African elephants (*Loxodonta africana*) and ~80 years for Asian elephants (Lahdenperä et al. 2014). Age-specific mortality and life expectancy have been presented for wild African elephants (Moss 2001; Gough and Kerley 2006; Foley and Faust 2010) and for working Asian elephants (Robinson et al. 2012; Hayward et al. 2014; Lahdenperä et al. 2014). Robinson et al. (2012) also examined the relationships between survival and reproductive output for their captive population and found positive covariance early in life where rapid reproduction was associated with higher survival, but negative trade-offs at older ages where reproduction increased mortality risks. In a global analysis of modern humans, Thomas et al. (2000) found that women who invested heavily in reproduction at younger ages had reduced longevity. Here, we explore whether similar trade-offs between reproductive rate and survival exist among wild female African elephants.

Given individual variation in susceptibility to mortality, mortality itself can alter the composition and vulnerability of older age classes (e.g. Vaupel and Yashin 1985). As Partridge (2001) and Carey and Judge (2001) discuss, survivors into old age may represent a specific class of organisms that remain after selective disappearance, rather than a random selection of heterogeneous individuals. However, if all young animals survive well, then the older cohorts will consist of more heterogeneous, less intensely selected individuals. Hawkes et al. (2012) relate these differences to *the varying strength of the mortality filters* (pg. 111). We ask whether we can detect mortality filters in a very long-lived species by assessing rates of reproduction in long-lived individuals by comparison to those that die young. Such longitudinal examinations of age-related changes in fecundity and survival should contribute to understanding the selective pressures shaping senescence patterns (van de Pol and Verhulst 2006; Monaghan et al. 2008).

Among reindeer (Weladji et al. 2010) and other ungulates (Loison et al. 1999; Gaillard et al. 2000), survival and rates of reproduction are lower and more variable in both young and older individuals compared to prime-aged females, and we assess these patterns for elephants. Older mothers are of particular interest. They may have fewer resources to allocate to reproduction due to depletion (e.g. Bérubé et al. 1999; Ericsson et al. 2001; Gaillard et al. 2003; Nussey et al. 2006; Weladji et al. 2010). Alternatively, in elephants where growth is prolonged, older, larger and more experienced females could allocate more time and milk energy to calves thereby ensuring offspring growth and survival (e.g. Clutton-Brock et al. 1984; Holand et al. 2006; Weladji et al. 2006, 2010), and such care allocation may be specifically required for the sons of older females (Lee and Moss 1986).

If the capacity to allocate care increases with age, older females are predicted to continue to reproduce into later life without either benefits or costs to daughters. However, since elephant family groups act as collaborative infant-rearing units (Moss and Lee 2011), older females might be expected to allocate more towards the survival of grandchildren at the expense of their own reproduction (e.g. Hawkes et al. 2011), especially if these females provide their offspring with a competitive advantage through knowledge transfer (Brent et al. 2015). Recent models of competition between related (mothers and daughters; Mace and Alvergne 2012) and unrelated females (mothers-in-law and daughters-in-law; Lahdenperä et al. 2012) with high generational overlap demonstrates fertility and survival costs to prolonged co-reproduction, and these costs contribute to selection for reproductive senescence and an extended lifespan. In such contexts, grandmothers are predicted to cease reproduction well before death. We therefore examine whether mothers who reproduce into older ages affect their co-resident daughters’ potential for reproduction, and whether possible grandmaternal contributions (direct or indirect) can occur while the grandmother remains reproductively active.

To address these questions, we describe age-specific reproductive output for 834 individually known wild African elephants in a 40+ year study period (representing ~60 % of a potential lifespan). Similar to moose (Solberg et al. 2004), elephants experience marked downstream growth, reproductive and survival consequences of a poor start in life (Lee et al. 2013; Mumby et al. 2015). We examine individual age-specific rates of reproduction in relation to a female’s early experience, age at first reproduction (e.g. Desprez et al. 2014) and longevity. We test an additional life history trade-off, recently documented for working Asian elephants: that early reproducers have higher age-specific fertility but shorter lifespans (Robinson et al. 2012). Finally, we explore the potential consequences of reproductive overlap between mothers and daughters.

## Methods

### Data collection

The study was conducted in and around Amboseli National Park, southern Kenya, from September 1972 and is on-going, with 42 years of observations analysed in this paper. The protected area covers 392 km^2^ while the ecosystem and the elephants’ range extends over approximately 8000 km^2^ (Moss et al. 2011). The population experiences minimal poaching across its range while hunting was outlawed in the 1970s. All members of the population are individually known and have been followed over the period of the study by means of a photographic recognition file or, in the case of young calves, by their association with known mothers. Individuals and family units (known kin units; Archie et al. 2006) are censused at least once per month for demographic events (births, deaths, oestrus, natal male independence). While some families can be elusive in the large ecosystem, the 60 known families were seen on average two to three times per month (range 0–6) (see Moss et al. 2011). A total of 44,676 independent sightings of elephants provide the baseline for demographic analyses.

The demographic dataset consists of birth dates assigned to all individuals (*N* = 2985; birth dates are coded with an accuracy of ±2 weeks, *N* = 2037; ±3 months, *N* = 332; ±6 months, *N* = 268; ±2.5 years, *N* = 243; ±5 years, *N* = 105). Almost 80 % of individuals are of known age. For those with estimated ages, age is relatively easy to evaluate from body and head dimensions since growth in height, back length and head circumference is prolonged into the 50s (Moss et al. 2011). Our visually assigned ages for older animals have been validated by comparisons with tooth ages after death using known-aged animals as a baseline (Lee et al. 2012). Death dates are based on dates of last known observation, carcass sightings, or records of illness/injury. Our long experience with the tight-knit structure of family units suggests that when a female has been missing from her family and not seen elsewhere for 2 weeks but her calves were present, she was dead. Accuracy of death dates was coded as for births with only 4 % of female deaths having a poorly known date. Maternity could be assigned for 2543 individuals, and a maternal age at each birth event and the interval between successive births (interbirth interval) was calculated.

### Population level survival

Age-specific survival was determined using Kaplan-Meier proportional hazards models for 1255 females with 511 natural deaths, including 178 females who entered the population with estimated ages (left censored) and 962 females with known ages. Survival was also assessed for a subset of 898 females that survived to age 9 (minimum age at first reproduction). Proportional hazards models were applied to females of known age giving birth to their first calf to determine age at first reproduction (AFR). We also examined calf longevity in relation to mother’s age at first reproduction for known-aged calves. Mortality rates are generally low in Amboseli, averaging 1.7 % of the total population in any year. Survival analyses considered only natural mortality although age-specific mortality rates did not differ between natural (*N* = 211) and human-caused deaths (*N* = 152) for females surviving to nine (Wald *χ*^*2*^ = 1.13, *p* = 0.287).

The use of hazard models assumes that the risks of mortality are similar for individuals entering the population after 1972 compared to those entering before 1972. We have no evidence for marked cohort differences between pre- and post-1972 females in age-specific survival rates, and the pre-1972 females entered at all ages ranging from a few months to 49 ± 5 years, so a specific cohort differential was not expected. The population of females was also right-censored, with 510 females in the reproductive sample still living at the end of 2014.

We report the mean and 95 % confidence intervals from hazards models for female survival and age at first reproduction for the population (*N* = 532 known-age females, with known ages at first birth).

### Event analyses for individual female reproductive rates

Each female who survived to 9 years was included in an individual event analysis (*N* = 834). Females with estimated ages were included only if they had a known reproductive trajectory over a minimum of 2 years (sufficient for one gestation length) within the 42 years of the study. Virtually all adult females in this population gave birth at least once and only three known-aged females have reached >20 years of age without giving birth. Of the females in annual fertility calculations, 82 % had known birthdates. Each year of life experienced by a female during the sample period (max *N* years = 41) was coded for birth/no birth event. Females entered and exited the longitudinal sample at different ages, as some were observed from <9 years to death (*N* = 196) or are still living (*N* = 504). Other females were only observed from after age 9 (*N* = 128 dead, 6 living). Since prior reproductive events were unknown for the 134 females who entered the sample over the age of 9, this left-censoring might influence individually based fertility calculations and we can only assume that the risk of an event at each age to females who joined the study after the age of first reproduction was unlikely to differ from those who joined at birth, similar to our left-censored survival rates above. If a female’s first reproductive event was unknown, she was not included in AFR analyses. Event analysis included all females irrespective of source of mortality since there were no age-specific biases in cause of death. We did not use the techniques of van de Pol and Verhulst (2006) because only 38.8 % of our females have a complete reproductive history terminating in death. As we have left- and right-censored data, we interpret our findings with caution.

The following variables were determined for each female: (a) her age at first birth (only for known first births); (b) calf probability of survival to 12 months and longevity of daughters for mothers which survived to that daughter’s minimum breeding age (9 years); (c) environment experienced in first year of life coded as drought (1) or no drought (0) (based on a drought severity index, see Lee et al. 2013); (d) age at death. Age at death was also coded into death before 40 years and survival to 40+, based on average longevity from hazard analysis. Maternal age was used in models as both a continuous and a categorical variable (young <20 as the maximum AFR, prime = 20–39, old >40 from average longevity). The use of categorical ages eliminates some of the potential contribution of ageing error to models.

The individual-based event analysis was also used to assess the duration of overlap between simultaneously breeding known mother–daughter pairs, which was compared with the daughter’s age-specific reproductive rate, following Mace and Alvergne (2012). Daughters’ reproductive rates were calculated as number of calves produced by each female for every 5 years (one interbirth interval) that she was present in the sample from ages 10 to 40, and then averaged across young and prime maternal ages to reduce variance introduced by intrinsically lower rates in younger mothers. Most known daughters were of prime reproductive age (mean ± SE years = 26 ± 0.5, *N* = 336). We used curve fit to determine the best fit relationship.

We used generalised linear mixed-models (GLMM) with a binomial distribution and logit link functions in SPSS 19 (IBM Corp. Chicago) to assess effects of maternal age, longevity, early drought, and age at reproductive onset on the probability of reproductive events occurring for each female. Age at each event was entered as a continuous variable, as well as age^2^ to improve what was obviously a non-linear fit. Fixed effects that did not contribute to the model were removed as long as the explanatory power of the model did not decrease. All two-way interactions were entered for initial models and then removed if they did not contribute to the overall model fit. We present final model *F* statistics, parameter estimates and coefficients (with 95 % Confidence Interval) for significant effects and interactions. Maternal identity was added to models as a random factor to control for repeated events for a female, although the variance contributed by identity was zero.

Calf survival to 12 months was also assessed using binomial logistic regression, with maternal age, birth order (first, not first, since maternal experience affects calf survival; Lee et al. 2013), drought and calf sex as fixed factors and maternal identity as a random factor. All data were tested for normality and log_10_ transformed if skewed. Interbirth interval (IBI) was log transformed for use in a generalised linear mixed-model, testing the effects of maternal age, calf sex, calf drought experience and birth order (first, not first) on IBI. Maternal identity was again entered as a random factor.

It was neither possible nor appropriate to record data blind because our study involved focal animals observed in the field and was based on known events.

## Results

### Survival, reproductive onset and reproductive rates

Mean longevity was 34 years (med = 37.9, 95 % CI = 35.1–40.7, *N* females = 1255, natural mortality only); 40 % of females born survived to age 40, 20 % to 50 and 10 % into their 60s. A female who survived to the minimum age at first reproduction (9 years) could expect to live on average for another 32 years (median = 41.25 years, 95 % CI = 39.7–42.8, *N* = 898), while a female who survived to 14 (average first reproduction, *N* = 758) would live for another 28 years. Cohort longevity or the age by which 95 % of females had died was 65 years.

**Fig. 1:**
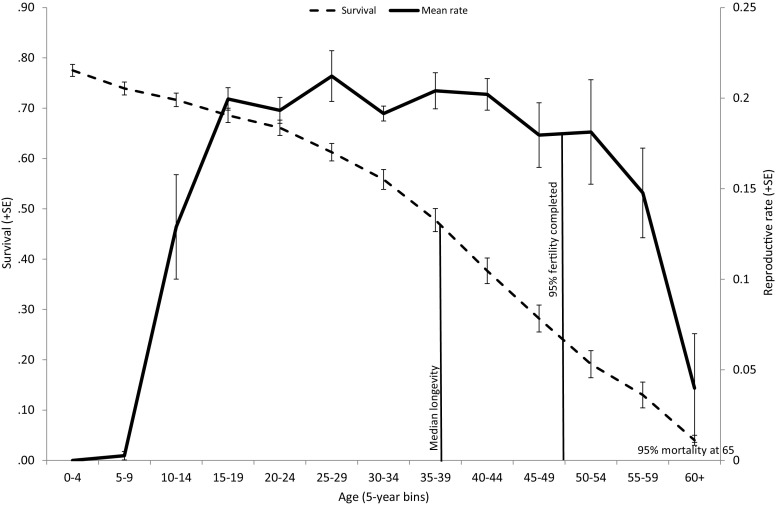
Population age-specific proportion surviving (±SE) in 5-year age bins for 1445 females from birth, plotted against mean (± SE) reproductive rates calculated from birth events for 834 females that survived to age 9 averaged over 5-year age bins (*N* females contributing: 639, 703, 637, 489, 379, 300, 231, 169, 115, 63, 33, 26, 12)

Mean known age at first birth was 13.86 years (median = 13.5, 95 % CI = 13.3–14.1, *N* = 532); early reproducers were subsequently classed as <12.5 (youngest quartile) and late reproducers classed as 15+ (oldest quartile). The age by which 5 % of females had commenced reproduction was 10.5 years. Once females commenced reproduction, they typically gave birth to a calf about every 5 years (mean = 0.186 births per female per year from 20 to 39—prime females), with a marked decline in reproductive rate after 40 (Fig. 1). The age where 95 % of cohort fertility had passed was 49 years. Contrary to expectations, variance in reproductive rates was lower for younger (0.133) and older (0.145) females than for the prime age group (0.159, Levene’s test for equality of variances, *F* = 65.52, *p* < 0.001).

A female’s age at first birth (early, average or late) was compared with her survival probabilities using hazard analysis. Early reproducers had similar longevity (mean = 36.8 years, 95 % CI = 33.7–39.8, *N* = 129) to late reproducers (mean = 39.6 years, 95 % CI = 37.4–41.7, *N* = 357, log-rank *χ*^*2*^ = 1.98, *p* = 0.16). Mothers who reproduced early were predicted to have offspring with reduced longevity, as they would be younger, smaller and potentially less able to invest in milk. However, offspring longevity did not vary as a function of their mother’s age at first reproduction (longevity for early AFR: mean = 17.2 years, 95 % CI = 14.8–19.3, *N* = 237; longevity for average AFR: mean = 23.5 years, 95 % CI = 19.9–27.2, *N* = 163; longevity for late AFR: mean = 20.3 years, 95 % CI = 17.4–23.2, *N* = 146; log-rank *χ*^*2*^ = 0.55, *p* = 0.76; known-age calves only).

**Fig. 2:**
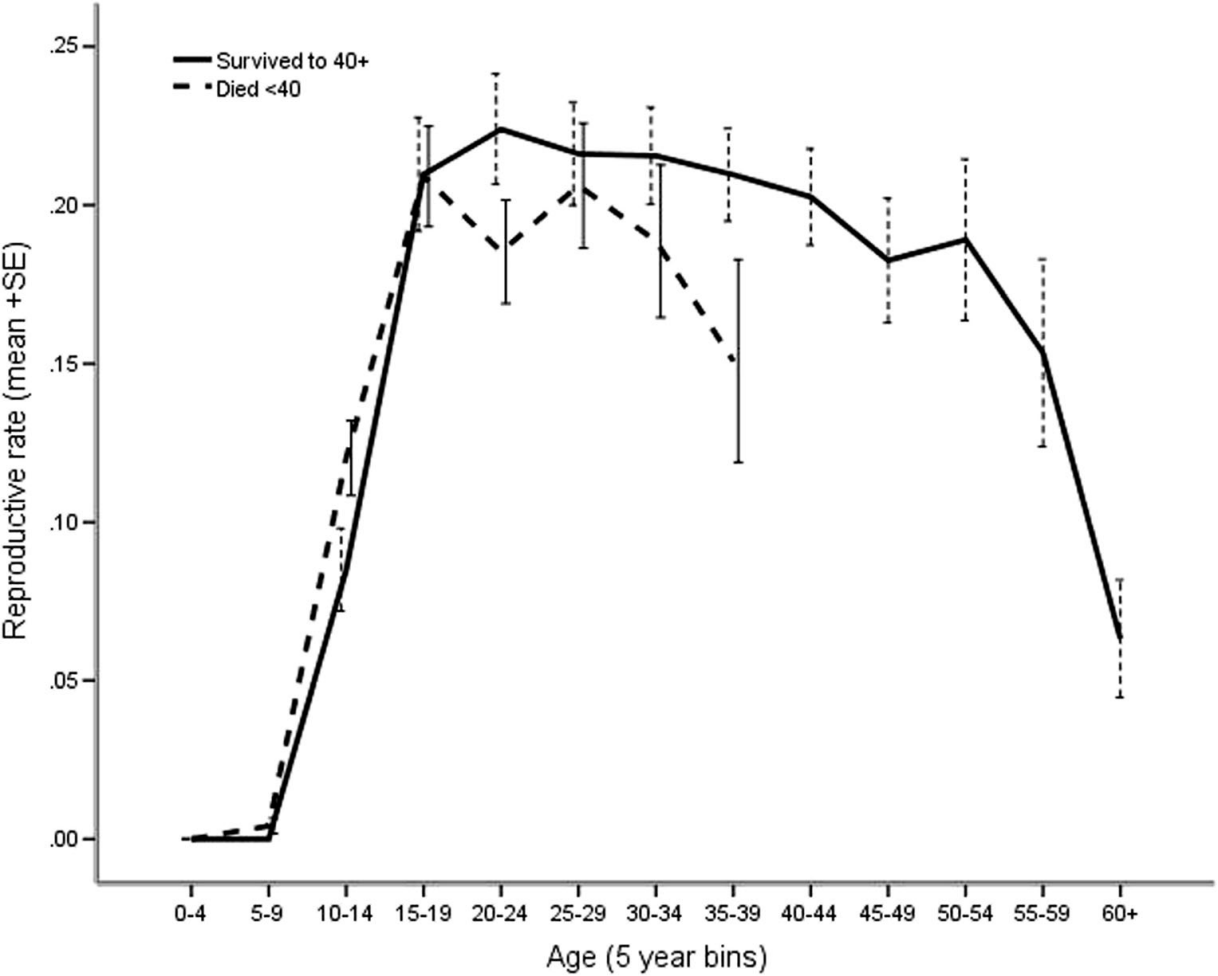
Age-specific reproductive events (±SE) for females who lived to 40+ (*N* = 165—*solid line*) by comparison with those who died before reaching 40 (*N* = 204—*dotted line*) (excludes 465 females yet to reach 40)

The probability of giving birth at each age was strongly related to age in a non-linear fashion (Table 1), with differences between survivors and those dying before 40 (Table 1, Fig. 2). Females commencing reproduction early (Table 1) had higher age-specific probabilities of giving birth. Reproductive rates for age were unaffected by whether the female’s age at entry to the sample was before or after age 9 (sample bias) or by her early drought experience.

**Table 1:** Binomial logistic regression for probability of a birth event by age, with overall model fit *F*, coefficient and estimated marginal means (95%CI) for significant fixed factors. Fixed factors (drought experience, age at entry) and interactions that did not contribute to the final model were excluded. Var_(ID)_ = 0.0000. Overall model explained 56.8 % of variance

	Main effect *F* and *p* value	Coefficient β (95 % CI) and *p* value	Estimated marginal means (95 % CI) age = 18.5; age^2^ = 520.47
Overall model fit	*F*_11, 19454_ = 23.67, *p <* 0.001		
Age	*F*_1, 19454_ = 172.78, *p <* 0.001	0.584 (0.502 to 0.593)	
Age^2^	*F*_1, 19454_ = 120.58, *p* < 0.001	−0.015 (−0.016 to −0.013)	
Age × age^2^	*F*_1, 19454_ = 75.95, *p* < 0.001	0.0000 (0.0000009 to 0.0000)	
Age at first reproduction (AFR)	*F*_3, 19454_ = 17.68, *p <* 0.001		
AFR early		2.009 (1.573 to 2.445), *p* < 0.001	0.038 (0.026–0.055)
AFR average		1.051 (0.676 to 1.426), *p* < 0.001	0.031 (0.022–0.044)
AFR late (reference)			0.021 (0.014–0.032)
Died before 40	*F*_2, 19454_ = 4.05, *p* = *0.018*		
Living, not yet 40		−0.130 (−0.256 to −0.005), *p* = *0.042*	0.020 (0.014–0.029)
Survived 40+		0.222 (0.088 to 0.356), *p* = 0.001	0.029 (0.020–0.041)
Died <40 (reference)			0.023 (0.016–0.034)
Interactions			
Age × AFR	*F*_3, 19454_ = 12.87, *p* < 0.001		
Age × AFR early		−0.076 (−0.097 to −0.056), *p* < 0.001	
Age × AFR average		−0.036 (−0.051 to −0.020)*, p* < 0.001	
Age × AFR late (reference)

### Age-specific investment in calves

Calf mortality in the first 12 months of life was unrelated to maternal age (*F*_1, 2418_ = 1.21, *p* = 0.30), although first-born calves (*F* = 5.73, *p* < 0.001; first = 0.204 (0.154–0.264), not first = 0.141 (0.154–0.264)), drought-born calves (*F* = 53.02, *p* < 0.001, dry = 0.243 (0.201–0.291), not dry = 0.116 (0.094–0.142)) and sons (*F* = 8.9, *p* = 0.003; males = 0.198 (0.163–0.238), females = 0.145 (0.117–0.179) were more likely to die in the first year, independent of maternal age (overall model fit: 86.6 %; *F*_5, 2418_ = 12.63, *p* < 0.001; variance_(ID)_ = 0.218 ± 0.117). The high contribution of individual to the model suggests that some mothers were consistently better or worse at rearing calves.

Interbirth intervals for mothers of calves that survived to 12 months differed significantly between young (mean = 47.6 months), prime (mean = 45.1 months) and old (mean = 48.4 months) mothers in the expected non-linear relationship (log_10_ IBI: GLMM overall model fit *F*_6,1520_ = 9.44, *p* < 0.001, age category: *F*_2,1520_ = 30.6, *p <* 0.001; variance (_ID_) = 0.001), and were longer for mothers of sons at all ages (*F*_1,1520_ = 4.54, *p* = 0.033, sons = 62.1 months, daughters = 48.5 months, mother’s age centred at 28.8 years). There was, however, no interaction between offspring sex and maternal age category (*F*_3,1250_ = 0.638, *p* = 0. 591). In addition, although sons required more time and energy than did daughters, no differences in birth sex ratio were found for mothers of any age (1286 F, 1281 M; binomial exact *χ*^*2*^ = 0.306, *p* = 0.86), nor for calves born to females aged over 40 (143 F: 153 M, *χ*^*2*^ = 0.338, *p* = 0.5).

### Grandmothering

Daughters with a mother that survived for at least her first 9 years of life had greater longevity (mean = 27.6 years, 95 % CI = 25.9–29.1) than did daughters whose mothers died before 9 years of age (mean = 18.9 years, 95 % CI = 16–21.1, log-rank *χ*^*2*^ = 45.9, *p <* 0.001, known-aged females only).

**Fig. 3:**
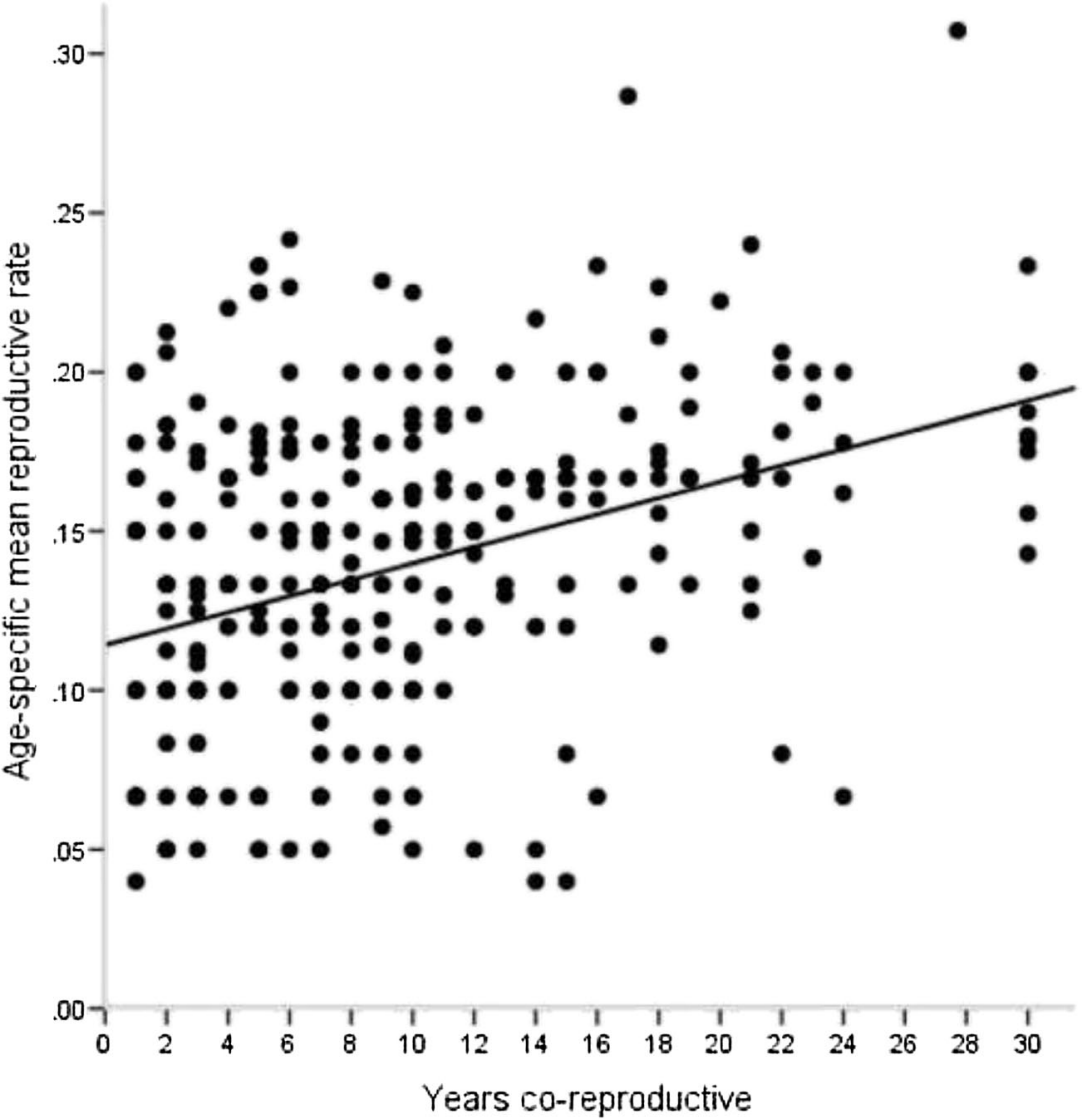
Age-specific rate of reproduction for individual females with a surviving mother (*n* = 336) as a function of the duration of overlap in breeding between known mother–daughter pairs (*β* = 0.003, constant = 0.114)

The time that known mother–daughter pairs were simultaneously breeding within their family unit ranged from 1 to 30 years and averaged 8.4 years. For females whose mothers were alive after they commenced reproduction, there was a slight enhancement in age-specific rates of reproduction with a longer duration of overlap between mother–daughter co-breeders (*r*^2^ = 0.135, *df* = 3, 321, *p <* 0.001; Fig. 3). Only 10 of 281 mothers survived for more than 10 years (two interbirth intervals) without giving birth to a calf while their daughters were also reproducing, suggesting that exclusive grandmothering in the absence of reproduction is not an elephant trait. In some large families, three generations of mother–daughter pairs could be simultaneously reproducing.

## Discussion

We used an individual-based approach for assessing age-specific fertility (Nussey et al. 2008; Robinson et al. 2012) and proportional hazards models for determining longevity among these known-age females. Amboseli elephant females exhibited the classical mammalian pattern of an age-specific decline in reproductive rate, but with moderately extended post-reproductive viability (Levitis et al. 2013; Lahdenperä et al. 2014). With an average fertility decline at 49 years for females, and cohort longevity of 65 years, the mean period of post-fertile viability (e.g. Levitis and Lackey 2011) was 16 years. Although the reproductive longevity of elephants appears to be greater than that of other non-human terrestrial species, this effect might be expected simply as a result of allometry (Carey and Judge 2001; Croft et al. 2015).

Less than 30 % of this population survived past the onset of fertility decline suggesting that survivors were unusual with retention of a small number of high viability individuals. Females who died before the median longevity of 40 years had reduced reproductive rates for their age while females who survived the *mortality filter* sustained higher rates of reproduction throughout their lives. These observed effects might represent the censored nature of the population with only 40 % of the study population having died. However, in their responses to a mortality filter, elephants are similar to humans (Thomas et al. 2000; Hawkes and Smith 2010), wild chimpanzees (Thompson et al. 2007) and a range of birds and mammals (Rebke et al. 2010) where longer survival is also associated with higher reproductive rates and reproductive senescence only becomes apparent at extreme old age. While we may be detecting the selective disappearance of poor reproducers in the population (e.g. van de Pol and Verhulst 2006), with low rates of mortality (as is characteristic of this population) even high-risk individuals may simply survive better (Hawkes et al. 2011).

Elephant annual fertility rates are constrained by a 22-month gestation as well as lactational anestrus of 12+ months, and their long lifespan encompasses numerous cycles of abundance and drought which potentially impact on their condition during reproduction. Elephant females also grow for at least the first 25–30 years of life, increasing in mass and height. After this age, they may still gain mass although only slowly (Hanks 1972). Thus, a female’s condition at each reproductive event is influenced by her early growth (Lee et al. 2013), the cumulative demands of the previous calf over its lactation period, which are more extreme for sons (see also Lee and Moss 1986; Moss and Lee 2011), and her current reserves and nutritional status (e.g. Wittemyer et al. 2007; Foley et al. 2008; Hayward et al. 2014). All of these factors could potentially influence the likelihood of an individual reproducing in addition to its age. Individual responses in reproductive potential, interacting with age-specific patterns of reproductive senescence, are seen in other long-lived species (Bérubé et al. 1999) and make it difficult to tease apart intrinsic vulnerabilities due to age from the consequences of local constraints.

Late age at reproductive onset, reflecting slow growth and poor physical condition early in life (Lee et al. 2013), was associated with a reduced probability of reproducing at each age. Early starters are significantly more productive in a variety of species (e.g. Nussey et al. 2006; van de Pol and Verhulst 2006; Robinson et al. 2012; Desprez et al. 2014) and the African elephant females in this sample who commenced reproduction early also had higher reproductive rates by comparison to later reproducers for the 40 year period of monitoring. Unlike working Asian elephants however (Robinson et al. 2012; Mumby et al. 2015), early reproducers appeared to suffer no marked survival consequences nor did their calves experience reduced longevity although, again, our sample is time-limited. Calf survival was a function of maternal experience and environmental conditions in the first year of life (see also Lee et al. 2013), but it was experience and not absolute maternal age that mattered. Unlike reindeer (Weladji et al. 2010) or Asian elephants (e.g. Lahdenperä et al. 2015), calf survival for older mothers did not differ significantly from that of prime females while the calves of primiparous females experienced the highest risk of early mortality. We found no evidence of differential investment at older ages: interbirth intervals for mothers of surviving calves followed a U-shaped curve, as would be expected given age-specific reproductive rates. Sons also appeared to be expensive for all females suggesting that there were no marked *end of life* sex-specific allocation strategies in these mothers. The lack of a trade-off between survival, reproductive onset and reproductive rate for age suggests positive covariance over the lifespan for the longer-lived females. We suggest that longevity has reproductive advantages, which are shared with family members through grandmothering, and thus an extended lifespan with prolonged fertility has been under positive selection.

The social advantages of longevity are clear for elephants; the oldest females act as *repositories of knowledge* (McComb et al. 2001, 2011; Mutinda et al. 2011), they actively *grandmother* calves providing protection and social cohesion even while also mothering their own calves, and early calf survival is enhanced by the presence of a grandmother in a family (Moss and Lee 2011). Only a small number of elderly females in this sample ceased reproduction prior to death with the potential to augment their daughters’ reproductive success through caring for grand-offspring (e.g. Mace and Alvergne 2012), while the presence of mothers even when reproducing themselves enhanced daughters’ reproductive rates. Maternal longevity within families thus improved daughter survival to reproductive age and appeared to increase rather than diminish their daughters’ reproductive rate, as predicted for female-kin units where grandmothers contribute to information exchange (Croft et al. 2015). Behavioral ageing, associated with cognitive declines or social inadequacies, therefore appears to be rare in elephants, at least until extreme old age, while reproductive senescence follows the age-specific pattern expected for a mammal of this size.
